# 1,1'-Carbonyldiimidazole-copper nanoflower enhanced collapsible laser scribed graphene engraved microgap capacitive aptasensor for the detection of milk allergen

**DOI:** 10.1038/s41598-021-00057-4

**Published:** 2021-10-21

**Authors:** Indra Gandi Subramani, Veeradasan Perumal, Subash C. B. Gopinath, Norani Muti Mohamed, Mark Ovinis, Lim Li Sze

**Affiliations:** 1 Centre of Innovative Nanostructures and Nanodevices (COINN), Universiti Teknologi PETRONAS, 32610 Seri Iskandar, Perak Darul Ridzuan, Malaysia; 2 Department of Fundamental and Applied Sciences, Universiti Teknologi PETRONAS, 32610 Seri Iskandar, Perak Darul Ridzuan, Malaysia; 3 Mechanical Engineering Department, Universiti Teknologi PETRONAS, 32610 Seri Iskandar, Perak Darul Ridzuan, Malaysia; 4 Institute of Nano Electronic Engineering , Universiti Malaysia Perlis (UniMAP) , Kangar, 01000 Malaysia; 5 Faculty of Chemical Engineering Technology, Universiti Malaysia Perlis (UniMAP) , Arau, 02600 Perlis, Malaysia; 6 Medical Innovation Ventures Sdn. Bhd (Mediven), Gelugor, 11700 Penang, Malaysia

**Keywords:** Materials science, Nanoscience and technology

## Abstract

The bovine milk allergenic protein, ‘β-lactoglobulin’ is one of the leading causes of milk allergic reaction. In this research, a novel label-free non-faradaic capacitive aptasensor was designed to detect β-lactoglobulin using a Laser Scribed Graphene (LSG) electrode. The graphene was directly engraved into a microgapped (~ 95 µm) capacitor-electrode pattern on a flexible polyimide (PI) film via a simple one-step CO_2_ laser irradiation. The novel hybrid nanoflower (NF) was synthesized using 1,1′-carbonyldiimidazole (CDI) as the organic molecule and copper (Cu) as the inorganic molecule via one-pot biomineralization by tuning the reaction time and concentration. NF was fixed on the pre-modified PI film at the triangular junction of the LSG microgap specifically for bio-capturing β-lactoglobulin. The fine-tuned CDI-Cu NF revealed the flower-like structures was viewed through field emission scanning electron microscopy. Fourier-transform infrared spectroscopy showed the interactions with PI film, CDI-Cu NF, oligoaptamer and β-lactoglobulin. The non-faradaic sensing of milk allergen β-lactoglobulin corresponds to a higher loading of oligoaptamer on 3D-structured CDI-Cu NF, with a linear range detection from 1 ag/ml to 100 fg/ml and attomolar (1 ag/ml) detection limit (S/N = 3:1). This novel CDI-Cu NF/LSG microgap aptasensor has a great potential for the detection of milk allergen with high-specificity and sensitivity.

## Introduction

Food allergy incidence is on a rising-trend and affect millions of people worldwide in-line with increased consumption of packed food. Cross-contamination and undeclared labelling are often the reasons behind food-allergic incidence, including skin rash, diarrhoea, vomiting, swollen limps, or even deadly anaphylaxis. A ready to use allergen detection kits can be beneficial to food manufacturers and consumers^1^. Milk is a vital food consumed by infants to elders and is the main ingredient in most food preparation^2^. However, β-lactoglobulin, which is the dominant bovine milk allergenic protein, induces an allergenic reaction to vulnerable individuals, especially in children, even in minute amounts^3^. A threshold value at 0.1 mg/ml was established to safeguard 99% of milk allergenic people and the International Dairy Federation (IDF) have proposed minimum permissible content of β-lactoglobulin for different type of milk: 2600 mg/l for pasteurized, 2000 mg/l for high-pasteurized and 50 mg/l for UHT^4,5^.

Biosensors are appropriate alternatives to conventional analytical methods such as ELISA, lateral flow immunoassay and mass spectroscopy, by offering comparable detection limit down to the attomolar range with high sensitivity and selectivity^6^. Various biosensors have been reported recently for the detection of β-lactoglobulin, including Surface Plasmon Resonance, microcantilever resonator array, faradaic-based electrochemical and colorimetric analyses with good detection limit (Table 1). However, most of them use expensive commercial electrodes such as ITO, silicon, GCE, and Screen Printed Graphene Electrode (SPGE) to construct the biosensor^7–12^. Recently, Laser Scribed Graphene (LSG) has become a popular approach to fabricate in-situ graphene directly into a desirable electrode pattern on a flexible substrate (PI/kapton, carbon paper). It is an excellent alternative to conventional fabrication, which require multiple processing steps, high-tech equipment and toxic chemicals. The use of LSG in biosensing for detecting various target entities has been reported^13–19^.

**Table 1:** Summary of available biosensors for β-lactoglobulin detection.

Transduction Technique	Probe	Nanomaterial	Electrode	Linear range	LOD	References
Faradaic voltammetry	DNA aptamer	–	Graphene-modified screen-printed carbon (GSPE)	100 pg/ml–100 ng/ml	0.02 ng/ml	^9^
Microcantilever resonator array	Antibody	−		0.1, 1, 10, 100 ppm	40 ng/ml	^10^
Photoelectrochemical (PEC) immunosensing	Antibody	Ag_2_S-sensitized spindle-shaped BiVO_4_/BiOBr heterojunction	ITO electrode	10 pg/ml–100 ng/ml	0.0037 ng/ml	^7^
Faradaic EIS	DNA aptamer	Flower-like BiVO_4_ microspheres	ITO electrode	0.01–1000 ng/ml	0.007 ng/ml	^8^
Faradaic voltammetric immunosensing	Antibody	−	Graphene-modified screen-printed carbon (GSPE)	1 pg/ml–100 ng/ml	0.00085 ng/ml	^12^
Surface plasmon resonance (SPR)			SPR sensor chip	0.49–1000 µg/ml	164 ng/mL	^45^
Colorimetric	DNA aptamer	Gold nanoparticles (AuNP), Graphene oxide	Paper	25 nM–1000 nM	12.4 nM	^11^
Non-faradaic capacitive aptasensing	DNA aptamer	CDI-Cu NF	LSG	1 ag/ml–100 fg/ml	1 ag/ml	This work

A biosensor's performance is often enhanced with the integration of nanomaterial structures from 0 to 3D. In this regard, multifunctional hybrid nanostructures have become increasingly popular and were applied successfully in diverse applications^20^. The use of organic–inorganic hybrid nanomaterials with 3D flower-like morphology are prevalent since its first discovery in 2012 by Zare et al.^21^. The hierarchical flower-like morphology with higher specific surface area, excellent catalytic activity, higher loading efficiency and strong adsorption capacity are the promising factors spurring its rapid development. Various inorganic (Cu^2+^, Ca^2+^, Zn^2+^, Mn^2+^, Mg^2+^, Fe^2+^, Co^2+^, and Ag^+^) and organic components (enzyme, aptamer, DNA, amino acids, peptides, antibody, raw chicken egg white, plant extract, curcumin, etc.) have been used to synthesize the hybrid nanoflower (NF) with different morphologies (rosette, rhombic, red blood cell-like, spherical)^22–28^.

1,1′-Carbonyldiimidazole (CDI) is an organic chemical with highly reactive carboxylating agent that contains two acylimidazole leaving groups with hydroxylated surfaces for bioconjugation of an analyte and often used as a linker in most biosensor surface construction^29–31^. The imidazole functional groups of CDI exhibit a strong interaction with the copper atom, forming charge-transfer complexes^32^. For the first time, CDI was used as an organic element with copper to synthesize a novel hybrid NF and used as a support for immobilizing oligoaptamer targeting β-lactoglobulin. The larger specific surface area of CDI-Cu NF allows a larger number of biomolecular adsorption that acts as a signal amplifier. The construction of the aptasensor starts with the fabrication of LSG with microgaps. A pair of a triangle-shaped electrodes were designed side by side with micrometre separation and was engraved in the form of graphene on flexible polyimide (PI) substrate via one-step CO_2_ laser irradiation. The electrode was then masked by a pre-designed laminating plastic film to expose only the biorecognition and electrode probing regions. The circular-shaped plastic lamination at the triangular junction acts as a reservoir, which reduces the size and overflow of a sample that could affect the sensor's performance. Next, the CDI-Cu NF was deposited at the pre-modified PI triangular junction of LSG microgap, followed by modification with neutravidin, oligoaptamer and finally, the detection of β-lactoglobulin.

## Results and discussion

LSG microgap electrode was fabricated via a simple and rapid one-step CO_2_ laser irradiation method and was masked by the lamination technique, leaving the triangular junction exposed for surface functionalization and target detection. The circle-shaped void at the triangular junction acts as a reservoir that allows the surface modification to be done only at the specific area and avoids PBS or any other chemical involved from spreading or overflowing. This reduces the sample consumption and avoids uneven surface wettability that could influence the capacitance measurement. Moreover, the CDI chemicals are usually used to immobilize biological molecules on the transducer surface. In this study, CDI as an organic element was co-precipitated with copper (Cu) metal to synthesize a novel self-assembled organic–inorganic hybrid NF, which was used as support for the immobilization of aptamer to target β-lactoglobulin.

### HPM and 3D-profiler surface imaging

**Figure 1:**
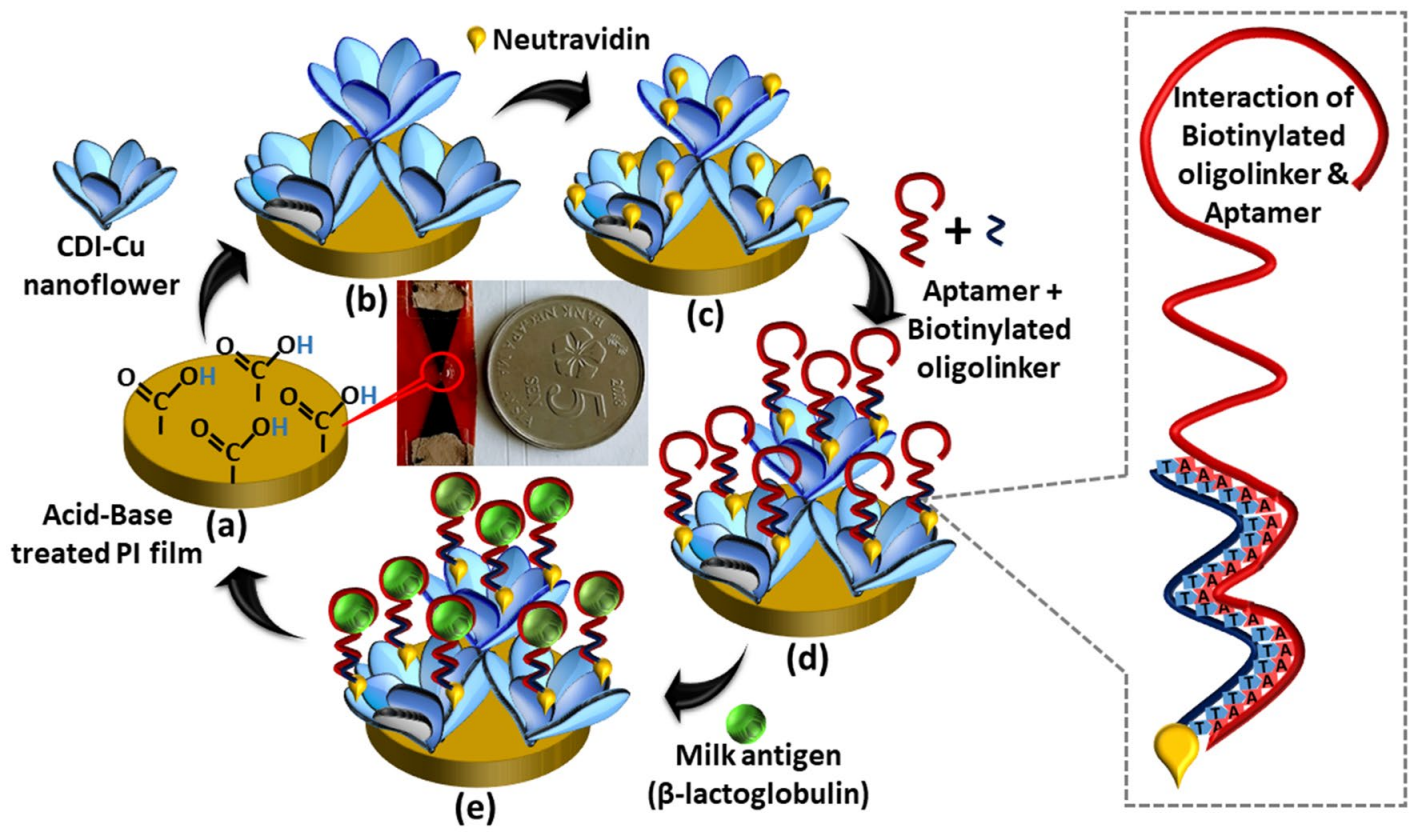
Stepwise fabrication of milk allergen, β-lactoglobulin biosensor on polyimide (PI) film, (**a**) acid–base treatment to terminate carboxylic functional group, (**b**) deposition of CDI-Cu hybrid NF, (**c**) modification with neutravidin, (**d**) immobilization of oligoaptamer, and (**e**) detection of β-lactoglobulin.

The morphology of the triangular junction with LSG microgap was investigated with 3D profiler and HPM (Fig. 1). The colour image of LSG microgap in Fig. 1a shows the variation in the height of the spongy-like structured LSG microgap and the scale at the right and bottom side represents the cross-section at the middle part of the LSG microgap. The trench or gap at the middle of two triangular electrode scribed side-by-side is clearly seen in the scale with a height variation, and greyscale image at Fig. 1b shows the 3D view of LSG microgap. Figure 1c shows the HPM image of LSG microgap under 5 × magnification with the circle surrounding the junction the reservoir pattern of the laminating film used to mask LSG microgap. Figure 1d shows the distribution of CDI-Cu NF around the triangular junction. The 3D architecture CDI-Cu NF offers a large specific surface area and abundant active sites for the immobilization of aptamer to target β-lactoglobulin.

### Field-emission scanning electron microscopy (FESEM): imaging, mapping and energy dispersive X-ray analysis (EDX)

**Figure 2:**
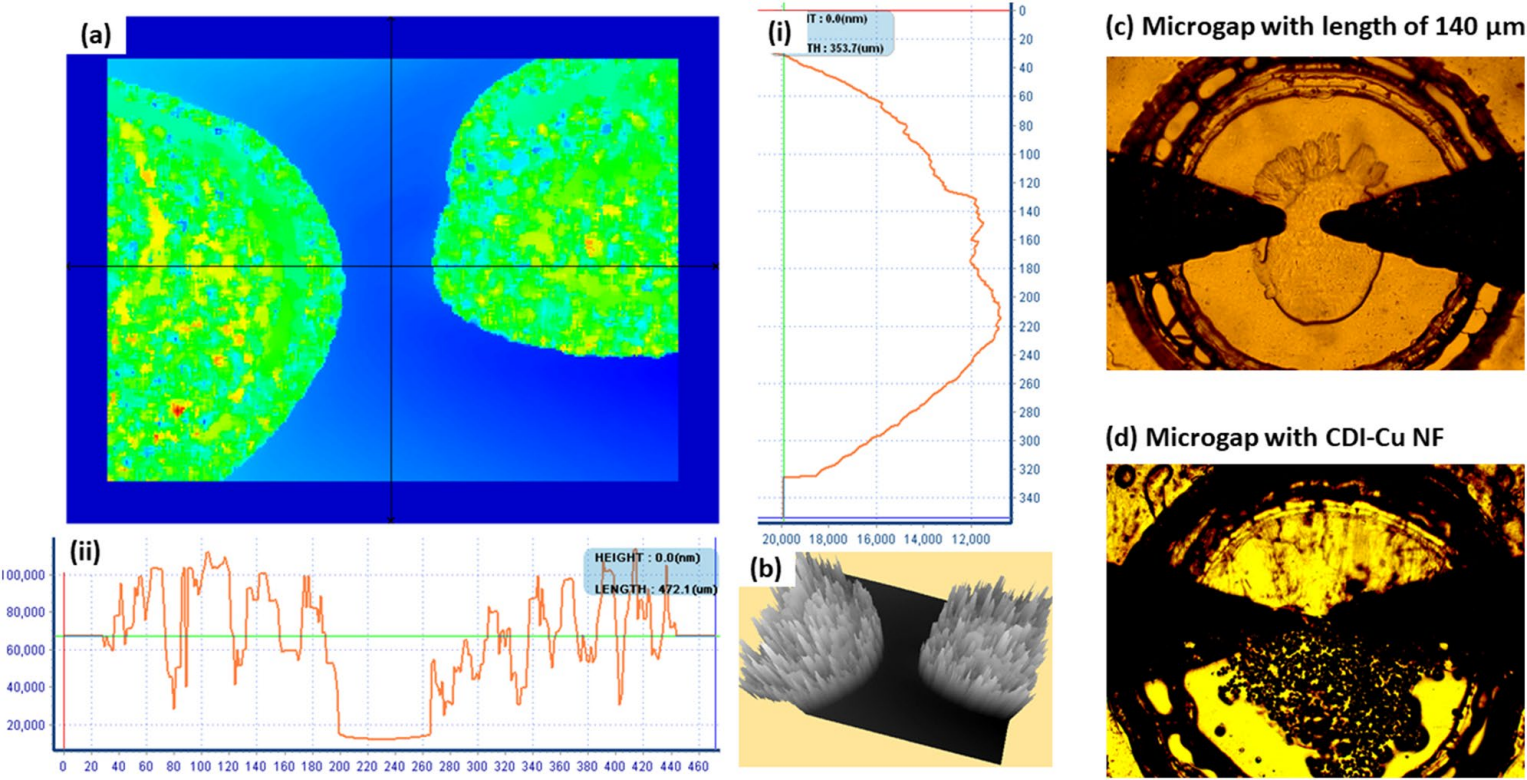
Morphology analysis of LSG microgap; (**a**) 2D color variation; (**b**) 3D image of LSG triangular junction; (**c**) HPM bare; (**d**) NF modified LSG microgap.

The morphology and the growing stage of CDI-Cu NF were investigated using FESEM, and the results are presented in Fig. 2. Figure 2 shows the FESEM images at various stages of NF synthesis, based on different concentrations and reaction times. Figure 2a–d shows the image of 0.05 mg/ml CDI-Cu NF with an incubation period of 1, 3, 5 and 7 days, respectively. Figure 2a shows the image of a bud-like structure, where NFs were in early developmental stages, while a whole floret with irregular petal sizes can be seen in Fig. 2c, with an incubation period of 5 days. Several undeveloped bud-like regions within the single NF can also be observed. With increasing incubation period, more defined complete flower-like morphology was seen (Fig. 2d) with multiple NFs of ~ 20–30 µm grown in a single branch. Increasing the concentration of CDI to 0.1 mg/ml significantly contributed to NF's growth. Figure 2e,f,g,h shows images of 0.1 mg/ml CDI-Cu NF with a different incubation period of 1, 3, 5, and 7 days. An excellent flower-like structure can be observed for 1-day incubation, and there are no significant morphology changes observed between 1 and 3 days of the incubation (Fig. 2e,f). However, more exposed petal structured NFs were aggregated in a single branch with a diameter of ~ 60–70 µm as the incubation was prolonged to 5 and 7 days (Fig. 2g,h). Supplementary Fig. 2 shows the elemental mapping and EDX image of CDI-Cu NF. Supplementary Fig. 2a–e shows a homogeneous distribution of significant elements including C, O, P, Cu and N, respectively. The presence of nitrogen was mainly contributed by the CDI chemical, and the mapping result supports the homogeneous distribution of the CDI element in the NF framework. The EDX analysis further confirms the presence of essential elements such as Cu, N, P, C, O within the NF (Supplementary Fig. 2f). The presence of element Au is from the sputtering procedure prior to FESEM analysis. The other elements, such as Cl, Na, and K, were endogenously attached from PBS solution used during the synthesis procedure.

### Chemical analysis by Fourier transform infrared spectroscopy (FTIR)

**Figure 3:**
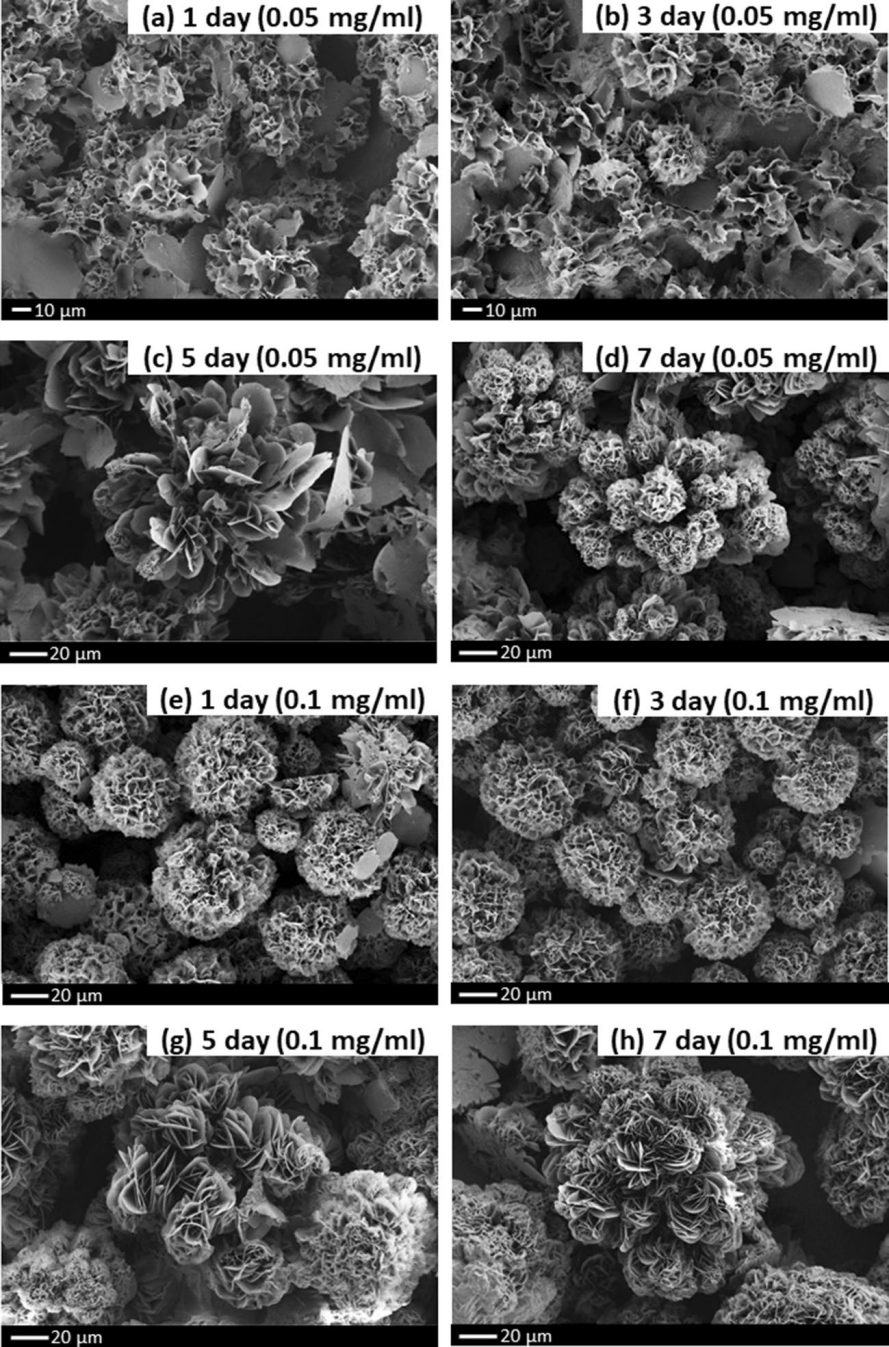
(**a**) FESEM image of CDI-Cu NF synthesized based on different concentrations and incubation periods. (**a**–**d**) 1, 3, 5, and 7 days of incubation for a CDI with 0.05 mg/ml, (**d**–**g**) 1, 3, 5, and 7 days of incubation for a CDI with 0.1 mg/ml.

The elemental composition changes upon PI film surface modification were monitored by FTIR analysis (Fig. 3). Figure 3a shows the FTIR spectra for CDI-Cu NF. The strong characteristic peaks at 630 cm^−1^ (bending) and 950–1100 cm^−1^ (symmetric and asymmetric stretching) were attributed to P–O and P=O vibrations, indicating the presence of copper phosphate group from the hybrid NF. Moreover, typical bands of organic element (CDI) for C-N were observed at 1363 cm^−1^ and for –CH at 2800–3000 cm^−1^^33^. Figure 3b represents the spectra for bare PI film exhibiting some prominent peaks including C-O bending (718 cm^−1^), C=O symmetrical stretching (1707 cm^−1^), C=O asymmetrical stretching (1777 cm^−1^), C–O–C asymmetrical stretching (1235 cm^−1^), C–O–C stretching (1088 cm^−1^), C=C aromatic ring (1500 cm^−1^), C–N stretching (1369 cm^−1^), N–H stretching (3624 cm^−1^)^34^. The modification with CDI-Cu NF concealed the PI film and weakened the PI film peaks (spectra c). Moreover, the sequential addition of layers (neutravidin, oligoaptamer) on the CDI-Cu modified PI film weakens the PI characteristics peak. When the oligoaptamer was immobilized on the CDI-Cu NF, stretching of (PO_4_)^3−^ (the sugar-phosphate backbone of DNA) was observed at 1082 and 1230 cm^−1^^35,36^. The hydrogen bonds of the DNA strands are notable from 3000 to 3500 cm^−1^^37^. Upon detection of target β-lactoglobulin, the peak of the amide is observed to be in the range of 1566–1742 cm^−1^.

### Capacitance analysis on surface modifications

**Figure 4:**
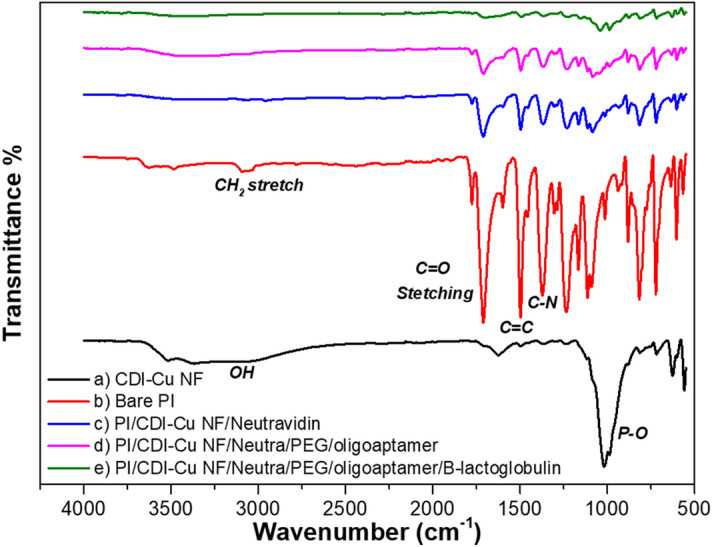
FTIR analysis of different modifications with PI film, (**a**) CDI-Cu NF; (**b**) bare PI film; (**c**) neutravidin/CDI-Cu NF; (**d**) oligoaptamer/neutravidin/CDI-Cu NF; (**e**) β-lactoglobulin/oligoaptamer/neutravidin/CDI-Cu NF.

All the non-faradaic capacitance measurements on the sequentially modified LSG microgap were carried out in 4 µl of PBS (10 mM, pH 7.4) to provide a neutral biological condition for the interactions of biomolecules. Any disturbance presented at the triangular junction with the separation of 95 ± 1.33 µm leads to a change in capacitance characteristics over a frequency range^35,38^. Figure 4a shows the capacitance measurement for different surface modified LSG microgaps. The bare LSG microgap exhibited a capacitance value in the pico-range (36.5 pF) while measurement in 4 µl of PBS increased the capacitance signal to the nano range (156 nF), due to the high permittivity of the PBS that contributes ions to the electrode. The capacitance value further increases threefold to a value of 460 nF with a distinctive signal after acid–base treatment. The acid–base treatment physically modifies the polyimide (PI) film at the triangular junction, terminating the carboxylic functional group. However, the deposition of CDI-Cu NF led to the drop of capacitance value to 426 nF. This is because the anionic natured NF with ~ 20–30 µm size has concealed the exposed acid–base modified PI film and is a barrier for the PBS in reaching the junction.

The capacitance value further reduced to 373 nF after modification with neutravidin. However, the application of PEG as a blocking agent increased the capacitance value to 404 nF and the immobilization of negatively charged oligoaptamer via Neutravidin-Biotin interaction increased the capacitance further to 523 nF. The highly specific and selective interaction of neutravidin and biotin forms a complex structure that increases the net molecular size at the triangular junction and affects the sensor's dielectric property. Changes in the charge distribution at the triangular junction leads to significant capacitance increment^39^. The biosensor's capacitance values increased gradually from 547 to 628 nF for the detection of a series of β-lactoglobulin with concentrations ranging from 1 ag/ml to 100 fg/ml. Higher concentrations of β-lactoglobulin were used to test the efficiency of the biosensor in sensing higher concentrations. The capacitance of 660 nF for 1 ng/ml was much more prominent than the signal obtained for a tenfold increase in concentration. The capacitance signal increases or decreases for each surface functionalization procedure obtained were similar for every tested sensor.

β-Lactoglobulin detection without CDI-Cu NF was also evaluated as well. The 0.1 mg/ml of CDI chemical was replaced after acid–base treatment on the LSG microgap (Fig. 4b). The modification by CDI chemical surged the capacitance signal from 316 nF (acid–base) to 439 nF, because the CDI chemical interacted effectively with the carboxylic acid terminated junction and allowed PBS diffusion (unlike CDI-Cu NF, which masks the junction and blocks PBS diffusion). An almost similar trend, in comparison with CDI-Cu NF modified sensor, was obtained for the modification with neutravidin and oligoaptamer immobilization. However, the signal is comparatively weaker for the detection of the various target concentrations. The relative changes in capacitance value from aptamer to target detection with and without CDI-Cu NF were 20.01% and 10.20%, respectively. The increment in the relative capacitance signal is associated with CDI-Cu NF, which provides larger specific surface area, and porous structure, both of which facilitates the effective capturing of target entities^33,40–44^. Figure 4c shows the impedance spectra signal is inversely proportional to the capacitance signal for every surface modification.

### Analytical performance of CDI-Cu NF modified sensor

**Figure 5:**
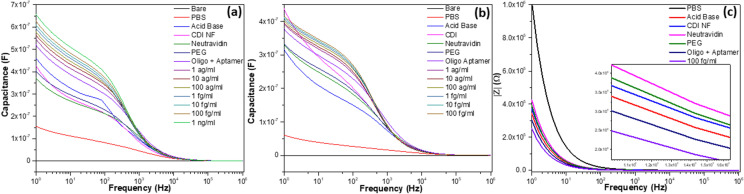
Capacitance analysis for detection of different concentrations of β-lactoglobulin. Tested from 1 ag/ml to 100 fg/ml with (**a**) CDI-Cu NF; (**b**) CDI chemical; (**c**) impedance spectra.

The reliability of the sensor was tested in terms of selectivity, reproducibility, and with spiked samples. Figure 5a shows the sensor's selectivity capacitance response, which was tested with 100 fg/ml of BSA and lysozyme. There are negligible changes in the response when tested with BSA and lysozyme, while there was significant increment in the response when tested with 100 fg/ml β-lactoglobulin, suggesting its efficiency towards the milk allergen detection. In addition, the developed biosensor was tested with 100 fg/ml β-lactoglobulin spiked food sample. Prior to the testing with the spiked food sample, the sensor was investigated using diluted food samples to reveal biofouling, as depicted in Fig. 5b. The biosensor was then tested with the 100 fg/ml β-lactoglobulin target spiked in 1:100,000 and 1:10,000 diluted food sample, as it gives the least deviation compared with the signal obtained for oligoaptamer. The spiked sample gives a significant capacitance response, suggesting the biosensors capability in detecting β-lactoglobulin in real food samples (Fig. 5c). Figure 5c inset represents the recovery table for 1:10,000 and 1:100,000 serum spiked targets which showed the recovery as 94.99% and 92.95% respectively.

Figure 5d shows a column chart for a batch of three biosensors for the detection of different concentrations of β-lactoglobulin. The three-column chart of devices 1, 2 and 3 represent a biosensor which was developed with LSG microgap electrode with almost identical separations of 96.82, 95.49, 94.16 µm, respectively. The relative standard deviation (RSD) value was estimated for the batch of three tested sensors with similar separations (Device 1, 2 & 3) in detecting a series of β-lactoglobulin concentrations ranging from 1 ag/ml (8.5%), 10 ag/ml (6.0%), 100 ag/ml (4.5%), 1 fg/ml (7.2%), 10 fg/ml (10.3%), and 100 fg/ml (9.1%).

Three biosensors (Device 1, 2 & 3) were used to obtain a calibration plot (Fig. 5e), which was used to analyse the limit of detection (LOD) and sensitivity of the biosensor. The relative capacitance change was calculated from the formula of (C_f_ − C_0_)/C_0_, where C_f_ and C_0_ represents the capacitance value at 1 Hz before and after exposure to the target β-lactoglobulin. The LOD of the developed biosensor is 1 ag/ml, estimated from calibration plot and experimental observation. The limit of detection (LOD) was considered the lowest concentration of an analyte (from the calibration line at low concentrations) against the background signal (S/N = 3:1). The sensitivity of the biosensor was 0.025 [(ΔC/C_0_)/(ng/ml)], calculated from the slope of the calibration plot (Fig. 5e). The stability of the sensor was analysed for seven days (Fig. 5f) where the sensor deteriorated 26.59%.

**Figure 6:**
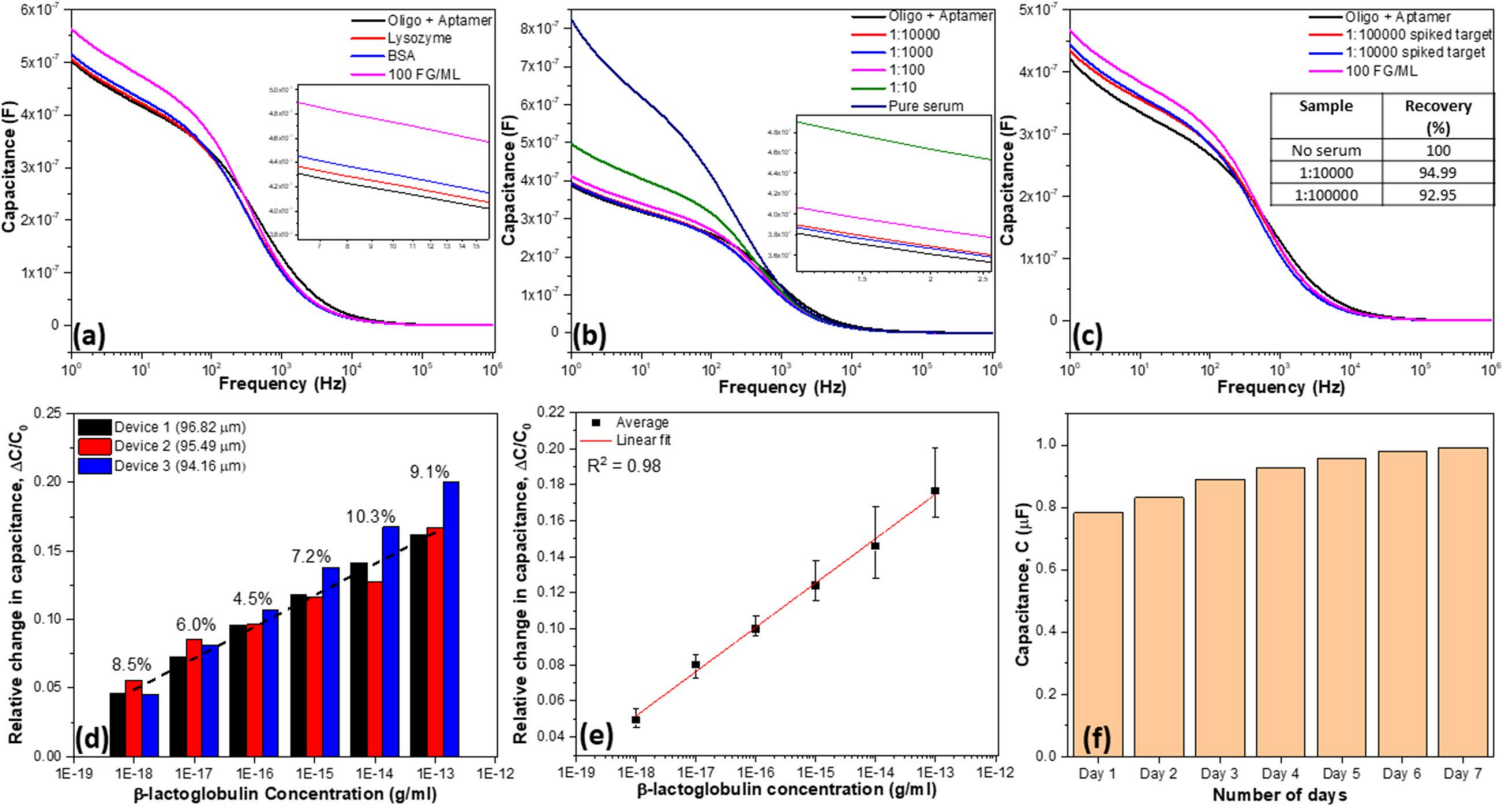
High-performance analysis. (**a**) Selectivity analysis; (**b**) food serum dilution factor analysis; (**c**) detection of spiked β-lactoglobulin and the inset represents the recovery results; (**d**) reproducibility column chart of three individual CDI-Cu NF/LSG microgaps. The separations of 96.82, 95.49, 94.16 µm with calculated RSD value for each concentration are ranging from 1 ag/ml to 100 fg/ml. (**e**) Calibration curve showing linear detection of β-lactoglobulin. (**f**) Stability column chart for seven days.

To complement the results obtained from the current sensing system, validations were performed by interacting β-lactoglobulin and aptamer using ELASA. The outcome of this experiment revealed the sensitivity of these molecular interaction is at 0.01 pg/ml with the linear concentrations range from 0.01 until 100 pg/mL and saturated at 100 pg/ml (Fig. 6).

The reported LOD is lower than commercial ELISA and other reported biosensors, with the ability to detect β-lactoglobulin in the attomolar range. Table 1 shows a summary of recently reported biosensors for the detection of β-lactoglobulin. A low LOD in attomolar range was achieved by incorporating LSG and 3D hierarchical NF. This simple and cost-effective fabrication technique is a promising approach for scalable fabrication and disposable sensors.

## Conclusion

A non-faradaic capacitive aptasensor enhanced by novel CDI-Cu NF modified LSG microgap was successfully developed for rapid and sensitive detection in the attomolar range of milk allergen, β-lactoglobulin. Aptamer was chosen for the specific recognition of the target. The careful design of the aptasensor ensured the low limit of detection at attomolar range, with good sensitivity and reproducibility, with the NF providing a unique high-performance sensing surface. Simple and cost-effective techniques were used in the preparation of the biosensor components. The electrode was prepared by a LSG technique, while the nanomaterials were prepared at room ambient by simple chemical mixing. This unique material composition has the potential for analysing a wide range of biomarkers for both clinical and non-clinical specimens.

## Methods

### Materials and chemicals

1,1′-Carbonyldiimidazole (CDI, reagent grade) and Copper sulphate pentahydrate were from Sigma Aldrich (USA). Phosphate buffer saline from 1st Base chemicals (PBS, 10 mM, pH 7.4) was used to synthesize CDI-Cu NF. Neutravidin procured from Thermofisher Scientific was deposited on the CDI-Cu NF modified electrode to aid the immobilization of oligo-aptamer. PEG was used as a blocking agent. β-lactoglobulin from Sigma Aldrich was used as a milk allergen target. Bovine Serum Albumin (BSA) and Lysozyme were used for selectivity analysis. Herbalife meal replacement shake from Formula 1 was used as a β-lactoglobulin free powder for the spiked sample analysis. Magnesium chloride (MgCl_2_) was used as a folding buffer to dilute the milk aptamer. Aptamer and biotinylated Oligolinker were purchased from Integrated DNA Technologies. PBS (10 mM, pH 7.4) was used for washing, diluting biomolecules, and as an electrolyte during the capacitance measurements. All figures are drawn by Microsoft PowerPoint 2016.

### Synthesis of CDI-copper nanoflower (CDI-Cu NF)

CDI-Cu NFs were synthesized in one pot^21^, with a slight modification. 20 µl of copper sulphate pentahydrate solution (200 mM) was added to a centrifugal tube containing CDI (3 ml, 0.1 and 0.05 mg/ml were prepared separately in PBS solution) and the mixture was vortexed at 3000 rpm for 30 s. Next, the mixture was allowed to rest for 24 h in room ambient. Blue-coloured precipitates were seen at the bottom of the centrifugal tube centrifuged at 4000 rpm for 15 min. The precipitates were filtered, washed thrice by Milli-Q water, and stored in a refrigerator at − 20 °C before use. A similar procedure was repeated with a different incubation times of 3, 5 and 7 days.

### Laser scribed graphene printing

Polyimide (PI) film was washed thoroughly with ethanol and dried in room ambient followed by irradiation with CO_2_ laser to engrave the graphene with the designed electrode pattern. Parameters include 30 W power at 50% of, speed of 30% and 500 PPI to print the capacitor-like LSG microgapped electrodes. The pattern of LSG with microgap was designated using CorelDraw software, and an array of electrodes was printed in every scribing procedure. The LSG microgap pattern consisting of a pair triangle was placed side by side with a sharp edge facing each other and separated by 95 µm. Finally, the electrodes were encapsulated using a previously patterned plastic laminating film to protect the contact point from the working electrolyte. The mask was patterned with a circular shape at the middle and square shape at both ends, the circular-shaped mask at the middle act as a reservoir for the working electrolyte. The reservoir helps to minimize the amount of chemical used during surface modification. The surface functionalization and electrochemical sensing were performed at the triangular junction of the electrode. The whole image of electrode is represented in Supplementary Fig. 1.

### Fabrication of aptasensor

**Figure 7:**
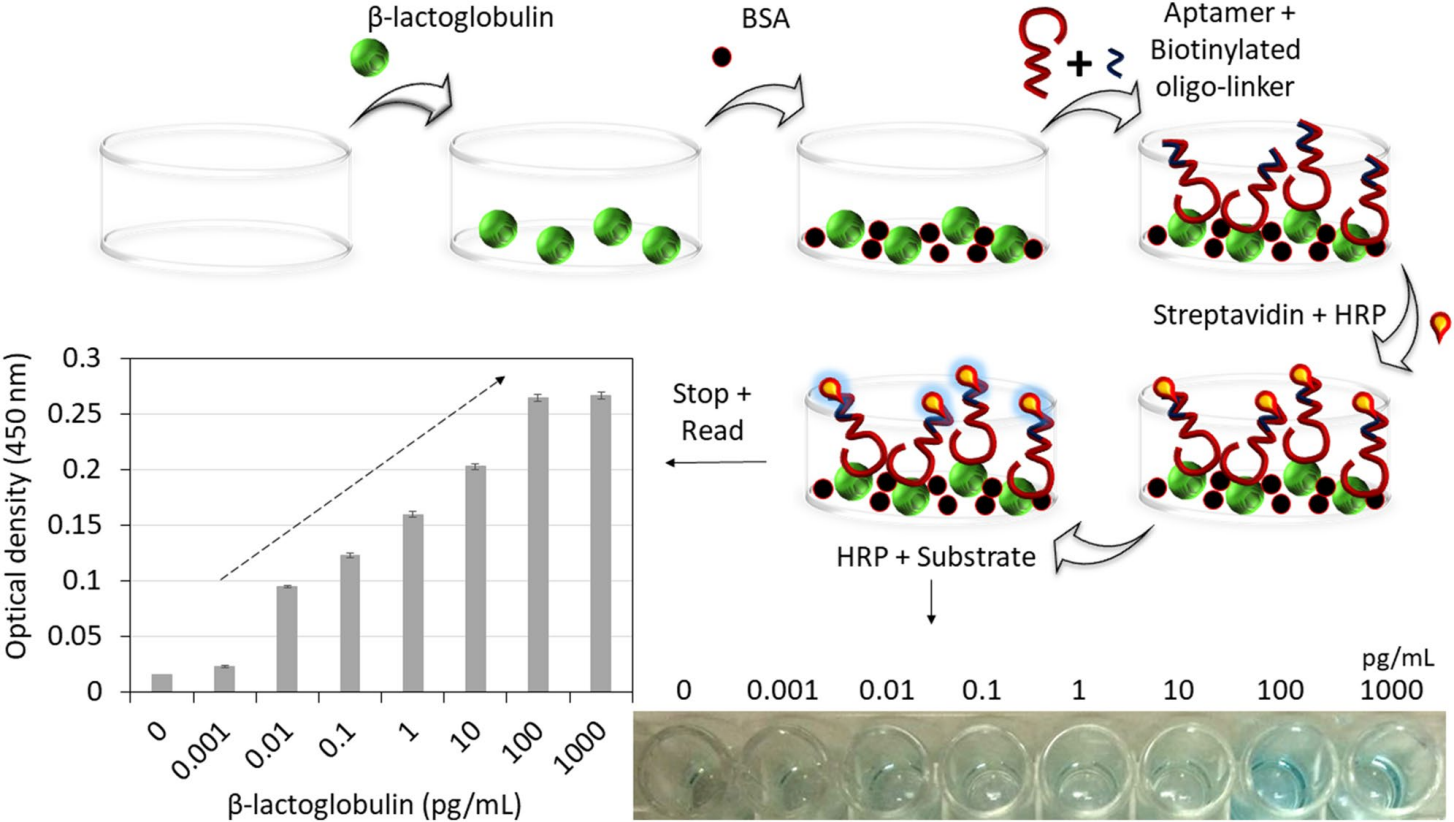
Validation by Enzyme-linked aptasorbent assay. Schematic image with the steps involved is shown. β-lactoglobulin concentrations from 0.001 until 1000 pg/ml were interacted with the constant aptamer concentration (50 nM). The results are displayed by bar-chart and arrow indicates the linear range. The photographed wells are shown as a figure-inset.

The PI film at the triangular junction of the bare LSG microgap was washed with PBS and subjected to further modification. First, the bare PI surface was incubated with 4 µl of 1 M Potassium hydroxide (KOH) for 10 min to activate the polyamic acid, followed by incubation with 4 µl of 1 M Hydrochloric acid (HCl) for another 10 min to functionalize the PI surface with carboxylic acid groups^32^. Next, the microgap (the separation) was pipetted with 0.5 µl of CDI-Cu NF and incubated for 1 h. Then, 4 µl of 0.1 mg/ml of neutravidin which were diluted in 10 mM PBS pH 7.4 was applied and incubated for 1 h. Next, 4 µl of 0.1 mg/ml of PEG was micro-pipetted and incubated for 15 min to cap the non-specific interaction sites. After that, 4 µl of β-lactoglobulin aptamer was pipetted and incubated for 1 h. Before application onto the microgap, the aptamer was diluted in a folding buffer (1 mM MgCl_2_, 10 mM PBS) and vortexed at 1000 rpm for 30 s, denatured at 90 °C for 5 min and mixed with biotinylated oligolinker. The oligolinker also acts as a spacer. Finally, a series of 4 µl of the β-lactoglobulin target was incubated for 10 min, and the capacitance was measured. The stepwise fabrication procedure of LSG microgap is illustrated in Fig. 7.

The biotin at one end of the oligolinker reacts with neutravidin through biotin-neutravidin interaction, while the other exposed free end of oligolinker with twenty thymine (T) bases readily bound with twenty additional adenine (A) bases modified milk aptamer, forming a strong bond. This spacer design (adenine & thymine bases modified on aptamer and biotin, respectively) was mainly carried out to ensure the aptamer is easily accessible by the target, as it was immobilized on CDI-Cu NF possessing a porous three-dimensional hierarchical structure.

Biotinylated Oligo-linker: 5′-/5Biosg/TTT TTT TTT TTT TTT TTT TT-3′.

β-lactoglobulin aptamer: 5′-ATA CCA GCT TAT TCA ATT CGA CGA TCG GAC CGC AGT ACC CAC CCA CCA GCC CCA ACA TCA TGC CCA TCC GTG TGT GAG ATA GTA AGT GCA ATC TAA AAA AAA AAA AAA AAA AAA AAA A-3′^11^.

### Spiked sample preparation

The food sample extract was prepared^9^ with a modification. 0.1 g of Herbalife meal replacement β-lactoglobulin free powder was mixed with 1 mL of PBS solution and shaken at 500 rpm for 1 h at 60 °C. The mixture was then centrifuged at 20,000×*g* for 15 min, and the clear supernatant was collected via micropipette and labelled as pure serum. The supernatant was diluted in PBS solution with different dilution factors (1:10,000, 1:1000, 1:100 and 1:10) to reveal biofouling. Finally, the dilution factor with the least deviation compared to the oligoaptamer capacitance signal was chosen to be spiked with 100 fg/ml of β-lactoglobulin and tested on CDI-Cu NF/LSG microgap biosensor at room ambient.

### Enzyme-linked aptasorbent assay (ELASA)

ELASA was carried out to validate the biomolecules involved in the developed β-lactoglobulin biosensor. β-lactoglobulin was diluted from 0.001 until 1000 pg/ml in 1X coating buffer and coated on an ELISA plate surface and was incubated overnight at 4 °C. Following a washing procedure by PBS, 2% of blocking agent, BSA was pipetted and kept for 1 h at room temperature. Next, 50 nM of β-lactoglobulin aptamer with biotinylated oligolinker was applied and incubated for 1 h. Prior to this step, 100 nM of anti-β-lactoglobulin aptamer was heated at 90 °C for 1 min and cooled to room temperature, added with equal volume of 100 nM oligolinker to obtain a final concentration of 50 nM and kept at room temperature for 10 min to make a duplex at the 3’ end of the aptamer. Followed that, 1:5000 diluted as-received HRP-Streptavidin conjugate was applied and held for another 1 h for the streptavidin to bind with biotinylated oligolinker-aptamer conjugates. TMB substrate was finally added and waited for 10–15 min for the colour change reaction. 50 μL of 2 mol l^−1^ sulfuric acid was added to each well to stop colour development. Finally, the optical density at 450 nm was used to measure the above solutions using UV-Nanodrop. PBS washing procedure was conducted thrice for every sequential step.

### Physical and electrical characterization

The morphology of synthesized CDI-Cu NF was investigated using variable pressure field-effect scanning electron microscope (VP-FESEM, Model: Zeiss Supra 55 VP), 3D-Nanoprofiler (Hawk 3D Optical Surface Profiler from Pemtron Co.Ltd., South Korea), and high-power microscope (Olympus, Japan). Elemental analysis of NF and different chemically modified surfaces were carried out using EDX and mapping method. The chemically modified different LSG surfaces were analyzed elementally using field-transform infrared spectroscopy (FTIR, Model: Perkin Elmer Spectrum One). All the capacitance measurements were carried out at room ambient using a standard two-electrode system (Alpha-A High-Performance Frequency analyzer, Novocontrol Technologies, Germany) in 4 µl PBS as a working solution. The frequency range was set with 1 to 100 kHz and Vrms at 10 mV.

## Supplementary Information

Supplementary Information.

## Data Availability

Relevant data included in the Supplementary Information.

## References

[1] Zhou J, Qi Q, Wang C, Qian Y, Liu G, Wang Y, Fu L (2019). Surface plasmon resonance (SPR) biosensors for food allergen detection in food matrices. Biosens. Bioelectron..

[2] Jiang D, Ge P, Wang L, Jiang H, Yang M, Yuan L, Ge Q, Fang W, Ju X (2019). A novel electrochemical mast cell-based paper biosensor for the rapid detection of milk allergen casein. Biosens. Bioelectron..

[3] Indyk HE, Hart S, Meerkerk T, Gill BD, Woollard DC (2017). The β-lactoglobulin content of bovine milk: Development and application of a biosensor immunoassay. Int. Dairy J..

[4] Lettieri M, Hosu O, Adumitrachioaie A, Cristea C, Marrazza G (2020). Beta-lactoglobulin electrochemical detection based with an innovative platform based on composite polymer. Electroanalysis.

[5] Boitz LI, Fiechter G, Seifried RK, Mayer HK (2015). A novel ultra-high performance liquid chromatography method for the rapid determination of β-Lactoglobulin as heat load indicator in commercial milk samples. J. Chromatogr. A.

[6] Montowska M, Fornal E, Piątek M, Krzywdzińska-Bartkowiak M (2019). Mass spectrometry detection of protein allergenic additives in emulsion-type pork sausages. Food Control.

[7] Sun X, Li C, Zhu Q, Huang H, Jing W, Chen Z, Kong L, Han L, Wang J, Li Y (2020). A label-free photoelectrochemical immunosensor for detection of the milk allergen β-lactoglobulin based on Ag2S-sensitized spindle-shaped BiVO4/BiOBr heterojunction by an in situ growth method. Anal. Chim. Acta.

[8] Xu S, Dai B, Zhao W, Jiang L, Huang H (2020). Electrochemical detection of β-lactoglobulin based on a highly selective DNA aptamer and flower-like Au@BiVO4 microspheres. Anal. Chim. Acta.

[9] Eissa S, Zourob M (2016). In vitro selection of DNA aptamers targeting β-lactoglobulin and their integration in graphene-based biosensor for the detection of milk allergen. Biosens. Bioelectron..

[10] Ricciardi C, Santoro K, Stassi S, Lamberti C, Giuffrida MG, Arlorio M, Decastelli L (2018). Microcantilever resonator arrays for immunodetection of β-lactoglobulin milk allergen. Sens. Actuators B Chem..

[11] Tah A, OlmosCordero JM, Weng X, Neethirajan S (2018). Aptamer-based biosensor for food allergen determination using graphene oxide/gold nanocomposite on a paper-assisted analytical device. BioRxiv.

[12] Eissa S, Tlili C, L’Hocine L, Zourob M (2012). Electrochemical immunosensor for the milk allergen Β-lactoglobulin based on electrografting of organic film on graphene modified screen-printed carbon electrodes. Biosens. Bioelectron..

[13] Ghanam A, Lahcen AA, Beduk T, Alshareef HN, Amine A, Salama KN (2020). Laser scribed graphene: A novel platform for highly sensitive detection of electroactive biomolecules. Biosens. Bioelectron..

[14] Lahcen AA, Rauf S, Beduk T, Durmus C, Aljedaibi A, Timur S, Alshareef HN, Amine A, Wolfbeis OS, Salama KN (2020). Electrochemical sensors and biosensors using laser-derived graphene: A comprehensive review. Biosens. Bioelectron..

[15] Xu G, Jarjes ZA, Desprez V, Kilmartin PA, Travas-Sejdic J (2018). sensitive, selective, disposable electrochemical dopamine sensor based on PEDOT-modified laser scribed graphene. Biosens. Bioelectron..

[16] Wan Z, Umer M, Lobino M, Thiel D, Nguyen NT, Trinchi A, Shiddiky MJA, Gao Y, Li Q (2020). Laser induced self-N-doped porous graphene as an electrochemical biosensor for femtomolar MiRNA detection. Carbon N. Y..

[17] You Z, Qiu Q, Chen H, Feng Y, Wang X, Wang Y, Ying Y (2019). Laser-induced noble metal nanoparticle-graphene composites enabled flexible biosensor for pathogen detection. Biosens. Bioelectron..

[18] Yagati AK, Behrent A, Beck S, Rink S, Goepferich AM, Min J, Lee MH, Baeumner AJ (2020). Laser-induced graphene interdigitated electrodes for label-free or nanolabel-enhanced highly sensitive capacitive aptamer-based biosensors. Biosens. Bioelectron..

[19] Hou L, Bi S, Lan B, Zhao H, Zhu L, Xu Y, Lu Y (2019). A novel and ultrasensitive nonenzymatic glucose sensor based on pulsed laser scribed carbon paper decorated with nanoporous nickel network. Anal. Chim. Acta.

[20] Kulkarni SK (2015). Nanotechnology: Principles and Practices.

[21] Ge J, Lei J, Zare RN (2012). Protein-inorganic hybrid nanoflowers. Nat. Nanotechnol..

[22] Zhang B, Li P, Zhang H, Fan L, Wang H, Li X, Tian L, Ali N, Ali Z, Zhang Q (2016). Papain/Zn3(PO4)2 hybrid nanoflower: Preparation, characterization and its enhanced catalytic activity as an immobilized enzyme. RSC Adv..

[23] Zhang T, Zhou Y, Wang Y, Zhang L, Wang H, Wu X (2014). Fabrication of hierarchical nanostructured BSA/ZnO hybrid nanoflowers by a self-assembly process. Mater. Lett..

[24] Rai SK, Narnoliya LK, Sangwan RS, Yadav SK (2018). Self-assembled hybrid nanoflowers of manganese phosphate and l-arabinose isomerase: A stable and recyclable nanobiocatalyst for equilibrium level conversion of d-galactose to d-tagatose. ACS Sustain. Chem. Eng..

[25] Ocsoy I, Dogru E, Usta S (2015). A new generation of flowerlike horseradish peroxides as a nanobiocatalyst for superior enzymatic activity. ACS Sustain. Chem. Eng..

[26] He L, Zhang S, Ji H, Wang M, Peng D, Yan F, Fang S, Zhang H, Jia C, Zhang Z (2016). Protein-templated cobaltous phosphate nanocomposites for the highly sensitive and selective detection of platelet-derived growth factor-BB. Biosens. Bioelectron..

[27] Zheng L, Sun Y, Wang J, Huang H, Geng X, Tong Y, Wang Z (2018). Preparation of a flower-like immobilized D-psicose 3-epimerase with enhanced catalytic performance. Catalysts.

[28] Jing M, Fei X, Ren W, Tian J, Zhi H, Xu L, Wang X, Wang Y (2017). Self-assembled hybrid nanomaterials with alkaline protease and a variety of metal ions. RSC Adv..

[29] Okutucu B, Telefoncu A (2004). Covalent attachment of oligonucleotides to cellulose acetate membranes. Artif. Cells. Blood Substit. Immobil. Biotechnol..

[30] Goddard JM, Erickson D (2009). Bioconjugation techniques for microfluidic biosensors. Anal. Bioanal. Chem..

[31] Yan M, Ge S, Gao W, Chu C, Yu J, Song X (2012). Fluorescence immunosensor based on P-acid-encapsulated silica nanoparticles for tumor marker detection. Analyst.

[32] Kim HJ, Park YJ, Choi JH, Han HS, Hong YT (2009). Surface modification of polyimide film by coupling reaction for copper metallization. J. Ind. Eng. Chem..

[33] Gandi I, Perumal V, Gopinath SCB (2020). 3D nanoporous hybrid nanoflower for enhanced non-faradaic redox-free electrochemical impedimetric biodetermination. J. Taiwan Inst. Chem. Eng..

[34] Kizil H, Pehlivaner MO, Trabzon L (2014). Surface plasma characterization of polyimide films for flexible electronics. Adv. Mater. Res..

[35] Qureshi A, Gurbuz Y, Niazi JH (2015). Chemical label-free capacitance based aptasensor platform for the detection of HER2/ErbB2 cancer biomarker in serum. Sens. Actuators B. Chem..

[36] Deshmukh R, Prusty AK, Roy U, Bhand S (2020). A capacitive DNA sensor for sensitive detection of on the Z3276 genetic marker: Fabrication. Analyst.

[37] Ramanathan S, Gopinath SCB, Arshad MK (2019). Aluminosilicate nanocomposite on genosensor: A prospective voltammetry platform for epidermal growth factor receptor mutant analysis in non-small cell lung cancer. Sci. Rep..

[38] Salila Vijayalal Mohan HK, Chee WK, Li Y, Nayak S, Poh CL, Thean AVY (2020). A highly sensitive graphene oxide based label-free capacitive aptasensor for vanillin detection. Mater. Des..

[39] Nunna BB, Mandal D, Lee JU, Singh H, Zhuang S, Misra D, Bhuyian MNU, Lee ES (2019). Detection of cancer antigens (CA-125) using gold nano particles on interdigitated electrode-based microfluidic biosensor. Nano Converg..

[40] Tang Q, Zhang L, Tan X, Jiao L, Wei Q, Li H (2019). Bioinspired synthesis of organic: inorganic hybrid nano flowers for robust enzyme-free electrochemical immunoassay. Nano Converg..

[41] Zhang Z, Zhang Y, Song R, Wang M, Yan F, He L, Feng X, Fang S, Zhao J, Zhang H (2015). Manganese (II) phosphate nanoflowers as electrochemical biosensors for the high-sensitivity detection of ractopamine. Sens. Actuators B. Chem..

[42] Zhang Z, Zhang Y, He L, Yang Y, Liu S, Wang M, Fang S, Fu G (2015). A feasible synthesis of Mn3(PO4)2@BSA nanoflowers and its application as the support nanomaterial for Pt catalyst. Catalyst.

[43] Zhao F, Bai Y, Cao L, Han G, Fang C, Wei S, Chen Z (2020). New electrochemical DNA sensor based on nanoflowers of Cu3(PO4)2-BSA-GO for hepatitis B virus DNA detection. J. Electroanal. Chem..

[44] Cao H, Yang D, Ye D, Zhang X, Fang X, Zhang S (2015). Protein-inorganic hybrid nanoflowers as ultrasensitive electrochemical cytosensing interfaces for evaluation of cell surface sialic acid. J. Electroanal. Chem..

[45] Ashley J, D’Aurelio R, Piekarska M, Temblay J, Pleasants M, Trinh L, Rodgers TL, Tothill IE (2018). Development of a β-lactoglobulin sensor based on SPR for milk allergens detection. Biosensors.

