# Supplementary material for: 1,1'-Carbonyldiimidazole-copper nanoflower enhanced collapsible laser scribed graphene engraved microgap capacitive aptasensor for the detection of milk allergen
Source: Sci Rep. 2021 Oct 21;11:20825. doi: 10.1038/s41598-021-00057-4 (PMC8531451; doi:10.1038/s41598-021-00057-4)
Supplement: Supplementary file 1 — Supplementary Information. [file 41598_2021_57_MOESM1_ESM.pdf]

Supplementary Information

1,1'-*Carbonyldiimidazole*-Copper Nanoflower Enhanced Collapsible Laser Scribed Graphene Engraved Microgap Capacitive Aptasensor for Detecting Milk Allergen

Indra Gandi Subramani[1,2], Veeradasan Perumal[1,3*],

Subash C.B. Gopinath[4,5,*], Norani Muti Mohamed[1,2], Mark Ovinis[3], Lim Li Sze[6]

[1]*Centre of Innovative Nanostructures and Nanodevices (COINN), Universiti Teknologi PETRONAS, 32610 Seri Iskandar, Perak Darul Ridzuan, Malaysia*

[2]*Department of Fundamental and Applied Sciences, Universiti Teknologi PETRONAS, 32610 Seri Iskandar, Perak Darul Ridzuan, Malaysia*

[3]*Mechanical Engineering Department, Universiti Teknologi PETRONAS, 32610 Seri Iskandar, Perak Darul Ridzuan, Malaysia*

[4]*Institute of Nano Electronic Engineering, 01000 Kangar,* [5]*Faculty of Chemical Engineering Technology, Universiti Malaysia Perlis, 02600 Arau, Perlis,* Malaysia

*Corresponding: veeradasan.perumal@utp.edu.my/subash@unimap.edu.my

Supplementary Figure 1: LSG MG electrode

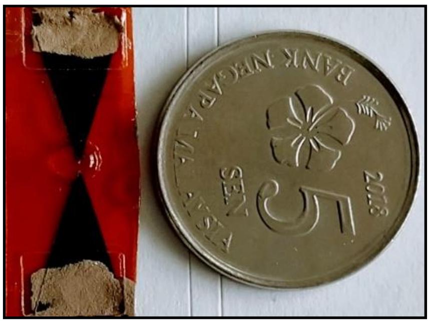

Supplementary Fig 1. LSG microgap design with a separation of ~95 µm. Two triangle shaped electrodes are placed side by side with the sharp edge facing each other. The plastic laminating film was pre-designed with an exposed circular area at the triangular junction for specific surface functionalization, and the exposed square-shaped area at both ends is for electrical probing.

Supplementary Figure 2: FESEM element mapping

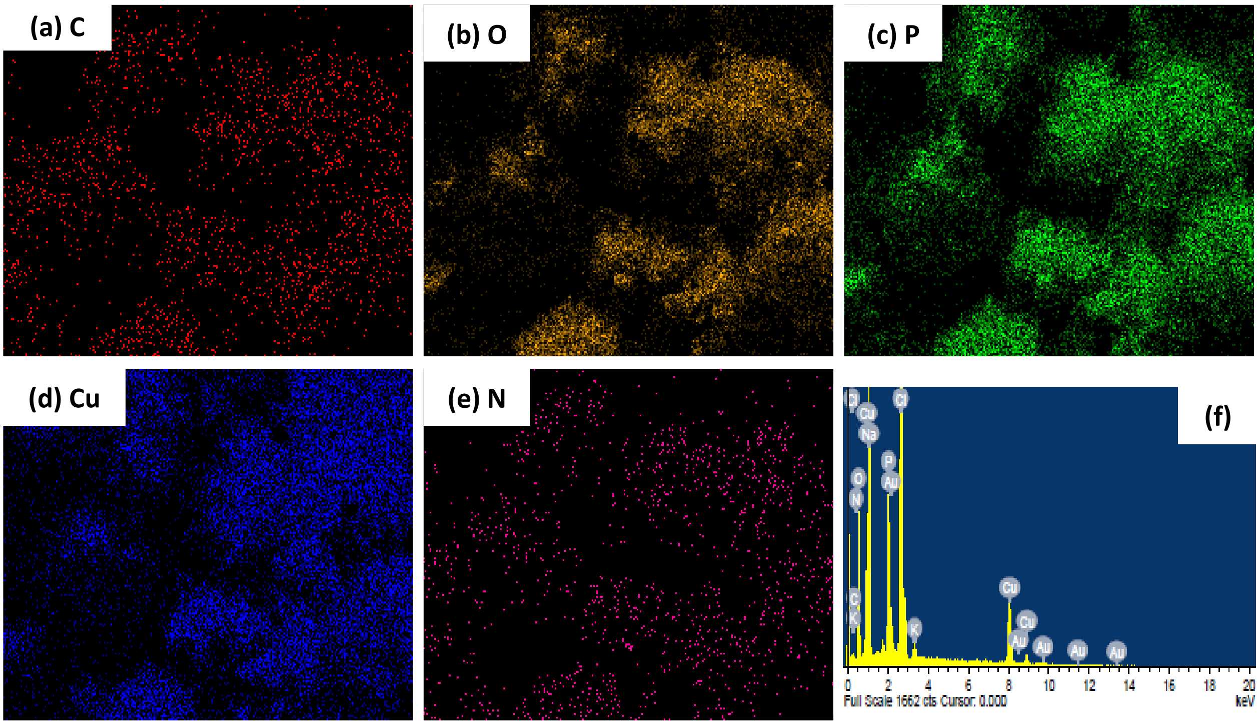

Supplementary Figure 2. (a, b, c, d, e) Mapping of elements C, O, P, Cu, N, and (f) EDX analysis of CDI-Cu nanoflower of 0.1 mg/ml concentration and 5 days of incubation period.
